# Factors related to severe single-vehicle tree crashes: In-depth crash study

**DOI:** 10.1371/journal.pone.0248171

**Published:** 2022-01-28

**Authors:** Kateřina Bucsuházy, Robert Zůvala, Veronika Valentová, Jiří Ambros

**Affiliations:** CDV - Transport Research Centre, Brno, Czech Republic; Tongii University, CHINA

## Abstract

Vehicle-tree collisions are the most common type of road crash with fixed obstacle in Czech Republic. Based on the literature review and using real world in-depth crash data, this paper aims to define factors, which significantly influence the injury severity of single vehicle-tree crashes. In-depth data provide a comprehensive view to the failure on the system infrastructure—human—vehicle related to crash, the in-depth crash database include very detailed information related to infrastructure, vehicle, human failure and crash participants characteristics and their medical condition and also crash reconstruction. Multinomial logistic regression and generalized linear mixed model were used to determine the individual effect of each predictor. The statistically significant variables were the day period, trunk diameter and impact speed. Using multinomial logistic regression shows also vehicle age as statistically significant. Obtained results can help to efficiently direct countermeasures not only on the road infrastructure—e.g. speed reduction in selected locations with specified tree character. However, the emphasis should be also focused on driver behaviour.

## Introduction

Vehicle-tree collisions are the most common type of road crash with fixed obstacle in Czech Republic. Analyses of vehicle crash tests as well as real world crashes show that impacts with rigid objects, such as trees can often lead to severe injuries (e.g. [1–3]). During tree collision vehicle decelerate heavily, which leads to significant vehicle overloading occurs, frequently followed by serious or fatal injuries. Therefore, trees in the road surroundings limit the road forgiveness. On the other hand, trees around roads may also have positive effect on driver behaviour and related driving speed e.g. Naderi et al. [4], Martens et al. [5], De Waard et al. [6], Shinar et al. [7], Edquist et al. [8], Burden [9]. Because of the high risk rate of the single vehicle collision with trees, the issue of the trees around roads is often considered in research studies. Particular attention is given to the frequency and severity of crashes in relation to the protection zone of the road.

### Literature review

There has been a number of factors which could influence the injury severity. Number of studies dealt with the effect of speed on the severity of injuries to crashes. Great attention was paid especially to vulnerable traffic users—especially pedestrians (e.g. [10–12]). Statistical models were often used to analyze factors which significantly influence injury severities (e.g. [13–15]). The analysis of rural single-vehicle crashes with fixed obstacles was conducted [16]. Among listed most dangerous ones were collisions with tree, shrubbery, utility poles or light poles. Impacts with trees are particularly prevalent as the most harmful event occurring in passenger car single-vehicle crashes [17]. Schneider et al. [18] analyzed driver injury severity resulting from single-vehicle crashes along horizontal curves. Trees were found to create the greatest increase in incapacitating and fatal injuries. As described by Naing et al. [1], mostly head and upper body have been injured as a result of impact of body and structures between A and B pillar. In this area, mostly the greatest level of intrusion occurred, especially when a rigid object intrudes directly into the vehicle occupant survival space. Based on in-depth crash analysis, Morris et al. [2] showed differences in injury outcomes which appeared to be related to the location of damage to the vehicle. As stated in Frampton et al. [19] narrow objects such as trees and poles are especially challenging for airbag deployments. Holdridge et al. [14] used multivariate statistical models of injury severity in fixed-object crashes, while accounting for roadway, vehicle, environmental, temporal, and driver characteristics. Fatal injuries were often associated with beam-guardrail leading ends, bridge rail leading ends, as well as tree stumps, light poles, utility poles, railway poles, traffic poles, overhead poles, and sign boxes.

Number of studies also analyzed the factors contributing to the roadside crashes or influenced injury severity. Bendigeri [20] based on the analysis of police crash reports suggested the possible correlation between the poor visibility (dark) condition and tree crashes occurrence. Zeigler [21] took into account driver characteristics (age and sex, influence of alcohol, residence of driver, time) and road environment (road type, rural/urban area, lane width, road marking, curvature, trunk diameter, distance of a tree). The dataset did not allow the speed determination. Daniello and Gabler [22] described the fatality risk of tree collision which was almost 15-times greater than the fatality risk of an overturn motorcycle collision. Cheng et al. [23] emphasized that the combination of steep side slope and small tree diameter should be studied, because errant vehicles tend to break the trees and roll-over.

Data from police statistics are commonly used for statistical analysis or modelling of the collision severity. These, however, cannot contain all the crashes characteristics. This could be the biggest limitation of the dataset as described e.g. by Wolf [24] during the analysis of the injury severity by crash type. In contrast, data from in-depth crash investigations contain information about the course of the crash and enable the comprehensive analysis of the injury mechanism in comparison to the vehicle damage as well as road infrastructure.

As described by Reed and Morris [17] there is a general lack of representative European crash data to aid the development of safety policy, regulation and technological advancement. The data are needed to both assess the performance of road and vehicle safety policies and to support the development of further actions by stakeholders. The tree crashes fatality requires a detailed analysis of traffic crashes and an understanding of the crash mechanism. The analysis of the most important factors affecting the severity of injuries could contribute to the targeted direction of measures for safe and forgiving road. Holdridge et al. [14] also stated that the analysis of crashes with roadside objects could provide integrated assessment of roadside hardware.

Van Treese II et al. [25] based on the literature review concluded the lack of studies that comprehensively analyze factors associated with increased risk of severe injury in tree crashes. In the previous studies only the selected limited number of factors are usually used. Therefore the aim of this paper was to identify factors, which significantly influence the injury severity, using in-depth data from real world crashes. As stated by Liu and Subramanian [26], the appropriate crash countermeasures based on identified factors can reduce the occurrence of the crashes and hence fatalities.

### Data

For the analysis, data from the research project Czech In-depth Accident Study (CzIDAS) was used, specifically data related to road crashes with injuries. The investigation team conducted the analysis immediately after crash occurrence at the crash scene. In-depth investigation includes participant interviews, detailed vehicle and infrastructure documentation and subsequent comprehensive analysis of the whole crash scenario including crash injury mechanism. Since CzIDAS project launch in 2011 more than 2000 traffic crashes have been analyzed. The data collection is based on the German In-Depth Accident Study (GIDAS) methodology.

The vehicle deformation and related injury severity could be influenced by various factors. The selection of tested variables was based on literature review and with respect to the dataset characteristics. Summary of selected research on tree crashes and impact of selected analysed parameters on crash severity and/or frequency is in Table 1. For the purpose of this study were tested all types of factors related to system infrastructure—crash participants—vehicle. For the modelling were also added some characteristics which were not widely used in previous studies—e.g. tree location or horizontal road marking which could influence driver perception and related driving speed. Impact speed is in some studies due to the data availability substituted by less precise speed limit or present suspicion on driver speeding (e.g. [18, 21]).

**Table 1:** Summary of selected research on tree crashes and impact of selected analyzed parameters on crash severity and/or frequency.

Analyzed parameters	Included in previous study
Driver age	Zeigler, 1986 [21]; Abidin et al., 2009 [27]; Bendigeri, 2009 [20]; Schneider et al., 2009 [18]
Impact speed	Holdridge et al., 2005 [14]; Wolf, 2006 [24]
Distance of a tree	Abidin et al., 2009 [27]; Zeigler, 1986 [21]; Turner et al., 1990 [28]; Lee and Mannering, 1999 [29]; Naderi, 2003 [30]; Dumbaugh, 2005 [31], 2006 [32]; Holdridge et al. 2005 [14]; Wolf, 2006 [24]; Mok et al., 2006 [33]; Park and Abdel-Aty, 2015 [34]; Armour et al. 1989 [35]
Tree trunk diameter	Turner et al., 1990 [28]; Zeigler, 1986 [21]
Vehicle age	Holdridge et al., 2005 [14]
Road width	Wolf, 2006 [24]; Zeigler, 1986 [21]
Road horizontal curvature	Schneider et al., 2009 [18]; Zeigler, 1986 [21]; Turner et al., 1990 [28]; Wolf 2006 [24]; Abidin et al., 2009 [27]
Day period	Zeigler, 1986 [21]; Turner et al., 1990 [28]; Abidin et al., 2009 [27]; Bendigeri, 2009 [20]
Driver gender	Zeigler, 1986 [21]; Wolf 2006 [24]; Holdridge et al., 2005 [14]; Schneider et al., 2009 [18]; Bendigeri, 2009 [20]
Belt usage	Holdridge et al., 2005 [14]; Schneider et al., 2009 [18]
Airbag activation	Schneider et al., 2009 [18]
Road type	Holdridge et al., 2005 [14]; Bendigeri, 2009 [20]
Location (rural/urban)	Zeigler, 1986 [21]; Wolf, 2006 [24]
Tree type (groups/isolated)	Dissanayake and Roy 2014 [36]
Vehicle mass	Holdridge et al., 2005 [14]
Inattention (yes/no)	Holdridge et al., 2005 [14]; Schneider et al., 2009 [18]
Fatigue	Schneider et al., 2009 [18]
Alcohol influence	Bendigeri, 2009 [20]; Zeigler, 1986 [21]; Holdridge et al., 2005 [14]; Schneider et al., 2009 [18]
Impact event type	Reed and Morris, 2012 [17]; Ray et al. 1991 [37]; Ray 1999 [38]
Passanger present	Schneider et al., 2009 [18]

The obtained dataset included 108 vehicle occupants injured during single-vehicle collisions with tree. The analyzed variables were selected with respect to the size of the dataset. For the modelling were used the injury suffered by all the vehicle occupants not only driver. The basic model included 7 continuous variables (Occupant age, impact speed, tree distance, tree trunk diameter, vehicle age, road width, vehicle mass) and 14 categorical variables (injury severity, horizontal curvature, road marking, day period, occupant gender, belt usage, road type, urban/rural area, tree location, inattention, alcohol influence, airbag activation, subjective traffic volume, impact type). Summary of the variables descriptive statistics show Tables 2 and 3.

**Table 2:** Summary of continuous variables.

	Mean	Median	Minimum	Maximum	Std. Deviation
Occupant age	35,64	31,00	3,00	84,00	17,39
Impact speed	52,25	48,00	10,00	104,00	20,73
Tree distance	332,18	330,00	15,00	1000,00	174,33
Tree trunk diameter	40,62	35,50	12,00	110,00	18,17
Vehicle age	13,04	13,50	1,00	25,00	5,83
Road width	678,07	620,00	280,00	2000,00	262,60
Vehicle mass	1281,99	1202,50	775,00	2200,00	298,21

**Table 3:** Summary of categorical variables.

		N	Marginal Percentage
Day period	day	79	73,1%
	night	29	26,9%
Occupant gender	male	63	58,3%
	female	45	41,7%
Alcohol influence	no	102	94,4%
	yes	6	5,6%
Belt usage	yes	91	84,3%
	no	17	15,7%
Horizontal curvature	straight	45	41,7%
	straight after curve	35	32,4%
	curve	28	25,9%
Impact event type	front	65	60,2%
	side	43	39,8%
Road type	1st class	12	11,1%
	2nd class	33	30,6%
	3rd class	56	51,9%
	Local roads	7	6,5%
Location	urban	9	8,3%
	rural	99	91,7%
Road marking	none	42	38,9%
	centreline only	44	40,7%
	both centreline and edgelines	22	20,4%
Inattention	no	101	93,5%
	yes	7	6,5%
Airbag activation	no airbag	27	25,0%
	activated	58	53,7%
	not activated	23	21,3%
Subjective traffic volume	random vehicles	68	63,0%
	slight traffic	35	32,4%
	heavy traffic	5	4,6%
Tree location	right	72	66,7%
	left	36	33,3%
Injury severity	slight	74	68,5%
	serious	17	15,7%
	fatal	17	15,7%

Prior to model development, exploratory analysis was conducted in order to check potential intercorrelations. The correlation analysis showed several significant correlations between following explanatory variables pairs: impact speed + occupant age, impact speed + vehicle age, impact speed + trunk diameter, tree distance from the road edge + road width. For categorical variables, the chi-square tests for all combination of categorical variables were used. The analysis shows the dependencies between some of the road environment characteristics. Dependent variables include road type, crash location (rural/urban), road marking, and tree location.

### Statistical modelling

Multinomial logistic regression could be used to determine the individual effect of each predictor. We assumed a vector of inputs (attributes) X = (*X*_1_, …, *X*_*p*_). The aim is to predict the output *Y* of the values 1, …, K. Predictor was denoted as G. Our goal is to classify the object described by attributes into one of the K classes. P(G = k|X = x) indicates the probability that the object described by the attribute vector x belongs to the class k. The logistic function is in the following form [39]:

$$logit\;\text{P}(\text{G}=\text{k}|\text{X}=\text{x})=\text{ln}\frac{\text{P}(\text{G}=\text{k}|\text{X}=\text{x})}{1-\sum_{l=1}^{K-1}P(G=l|\text{X}=\text{x})}$$

The transformed probability can be modeled similarly as for linear regression:

$$\begin{array}{c} logit\;P\left(G=1|\text{X}=\text{x}\right)=\beta_{1,0}+\beta_{1}^{\prime }x\\ logit\;P\left(G=2|\text{X}=\text{x}\right)=\beta_{2,0}+\beta_{2}^{\prime }x\\ \ldots \\ logit\;P(G=K-1|\text{X}=\text{x})=\beta_{\left(K-1,0\right)}+\beta_{\left(K-1\right)}^{\prime }x, \end{array}$$ (1)

where (*β*_1,0_, *β*_1_, …, *β*_*K*−1,0_, *β*_*K*−1_) are the parameters of the model. It follows that

$$\begin{array}{c} P\left(G=1|\text{X}=\text{x}\right)=\frac{\text{exp}\left(\beta_{1,0}+\beta_{1}^{\prime }x\right)}{1+\sum_{l=1}^{K-1}\text{exp}\left(\beta_{l,0}+\beta_{l}^{\prime }x\right)}\\ P\left(G=2|\text{X}=\text{x}\right)=\frac{\text{exp}\left(\beta_{2,0}+\beta_{2}^{\prime }x\right)}{1+\sum_{l=1}^{K-1}\text{exp}\left(\beta_{l,0}+\beta_{l}^{\prime }x\right)}\\ \ldots \\ P\left(G=K-1|X=x\right)=\frac{\text{exp}\left(\beta_{K-1,0}+\beta_{K-1}^{\prime }x\right)}{1+\sum_{l=1}^{K-1}\text{exp}\left(\beta_{l,0}+\beta_{l}^{\prime }x\right)}\;\text{for}\;K=2,\ldots ,k \end{array}$$ (2)

$$P\left(G=K|\text{X}=\text{x}\right)=\frac{1}{1+\sum_{l=1}^{K-1}\text{exp}\left(\beta_{l,0}+\beta_{l}^{\prime }x\right)}\;\text{for}\;\text{the}\;\text{reference}\;\text{category}\;\text{K}=1.$$ (3)

If the two variables are strongly correlated, then it is not possible to determine the independent influence of one of the variables on the result—multicollinearity. The high degree of multicollinearity is mainly due to the fact that the accuracy of the regression coefficient estimate is reduced. The cases when correlation coefficient exceeded 0.8 were considered a strong multicollinearity. The easiest way to eliminate or reduce the effect of multicollinearity is to remove one of the variables. However, some of the studies [40–43] prove that multicollinearity should not be used as a basis for not considering a variable in model estimation. Variable should be excluded after it has been found to produce a statistically insignificant parameter. The presence of strong correlation between explanatory variables does not cause any systematic bias of estimation as long as all the correlated variables are present in the model and the inferences are made within the region of observations. The only consequence of a strong correlation between variables in the model is sometimes a need for a larger sample to improve the model precision in estimating individual impacts. The problem arises when the standard errors of one or both of the correlated variables are high. The correlated variables were not removed from the dataset based on these findings and with regard to the characteristics of the correlated variables.

Generalized linear mixed models (GLMM) is an extension to the generalized linear model in which the linear predictor contains random effects in addition to the fixed effects. Fixed effects are the usual terms in the model. Fixed, because they have the same value for everyone in a group or subgroup. Random effects are error terms and anything else randomly chosen from some population. GLMM are particularly used when there is non-independence in the data. The general form of the model (in matrix notation) is [39]:

y=Xβ+Zu+ε (4)

Where ***y*** is the outcome variable; ***X*** is a matrix of the predictor variables; ***β*** is a column vector of the fixed-effects regression coefficients; ***Z*** is the design matrix for the random effects; ***u*** is a vector of the random effects; and ***ε*** is a column vector of the residuals.

### Logistic regression

Considering above, the logit model was created with all of the variables (including correlated variables). According to the regression parameters significance level (Sig.), the non-significant variables were excluded. The slight injury was selected as the reference category. The resulting model is significantly different from the null model (p-value = 0.000). The explanatory power of the model, in terms of Nagelkerke R2 is approximately 53%. The resulting model then contains significant variables: impact speed, trunk diameter, vehicle age, day period (The model results can be found in Table 4 and the estimated coefficient in Table 5).

**Table 4:** Likelihood ratio test.

	Chi-Square	Sig.
(Intercept)	.000	.
Impact speed	20.056	.000
Tree trunk diameter	10.694	.005
Vehicle age	7.513	.023
Day period	11.139	.004

**Table 5:** Parameter estimates.

Parameter		B	Std. Error	Sig	Exp(B)	95% confidence interval	
						Lower bound	Upper bound
Serious injury	Intercept	-6.867	1.733	.000			
	Impact speed	.043	.018	.017	1.044	1.008	1.081
	Trunk diameter	.055	.022	.010	1.057	1.013	1.103
	Vehicle age	.145	.062	.019	1.156	1.024	1.305
	day period = day	-1.496	.703	.033	.224	.056	.889
	day period = night	0^b^	.	.	.	.	.
Fatal injury	Intercept	-9.488	2.081	.000			
	Impact speed	.082	.022	.000	1.086	1.039	1.134
	Trunk diameter	.068	.024	.004	1.070	1.022	1.122
	Vehicle age	.137	.069	.046	1.147	1.003	1.312
	day period = day	-2.555	.855	.003	.078	.015	.415
	day period = night	0^b^	.	.	.	.	.

^a^. The reference category is: slight injury.

B—regression coefficient, Sig—achieved statistical significance, exp(B)—the effect of regression coefficients.

Standard errors are relatively small despite the correlation between impact speed and trunk diameter. If the variable impact speed increase about 10 kilometres per hour, the probability of serious injury increases 1.044^10^ = 1.54 times compared to the slight injury. In case of fatal injury, the probability increases 1.086^10^ = 2.28 times compared to the slight injury. Similarly, in case of tree diameter increase about 10 centimetres, the probability of serious increases 1.057^10^ = 1.74 times and probability of fatal injury 1.070^10^ = 1.97 times, compared to the reference category slight injury.

The vehicle age coefficient difference between serious and fatal injury are very similar. It could be assumed the increase of severe injury probability (fatal and serious) 1,15^10^ = 4,1times with increase in vehicle age about 10 years. The interpretation of the variable day period was reduced only to the signs of regression coefficient because of higher standard errors values of the categories of this variable. If the crash occurs at night, the probability of more severe injury has been higher compared to the day.

The resulting models may be rewritten as:

$$logit\;\text{P}\left(\text{G}=\text{serious}\;\text{injury}|\text{X}=\text{x}\right)=-6.867+0.043\cdot impact\;speed+0.055\cdot trunk\;diameter+0,145\cdot vehicle\;age+\{\begin{array}{c} -1.496\;for\;day\;period=DAY\\ 0\;for\;day\;period=NIGHT \end{array}$$ (5)

$$logit\;\text{P}\left(\text{G}=\text{fatal}\;\text{injury}|\text{X}=\text{x}\right)=-9.488+0.082\cdot impact\;speed+0.068\cdot trunk\;diameter+0,137\cdot vehicle\;age+\{\begin{array}{c} -2.555\;for\;day\;period=DAY\\ 0\;for\;day\;period=NIGHT \end{array}$$ (6)

If one of the correlated variables was removed (impact speed or trunk diameter), the model lost one statistically significant variable. If the trunk diameter has been removed, the power of the model (Nagelkerke R2 = 53%) decreased by approx. 7% (Nagelkerke R2 = 46%), in case of impact speed removal the power of the model decreased by approx. 14% (Nagelkerke R2 = 39%).

The statistically significant variables were similar in all of these three models—vehicle age, day period and trunk diameter or impact speed. Also, the coefficient values with exception of one of the correlated values were very similar as in the first model with all variables. Comparison of coefficient and standard errors between models showed negligible differences between estimated values. The removal of one significant variable would cause loss of one significant variable with concurrent decrease of power of the model.

**Fig 1:**
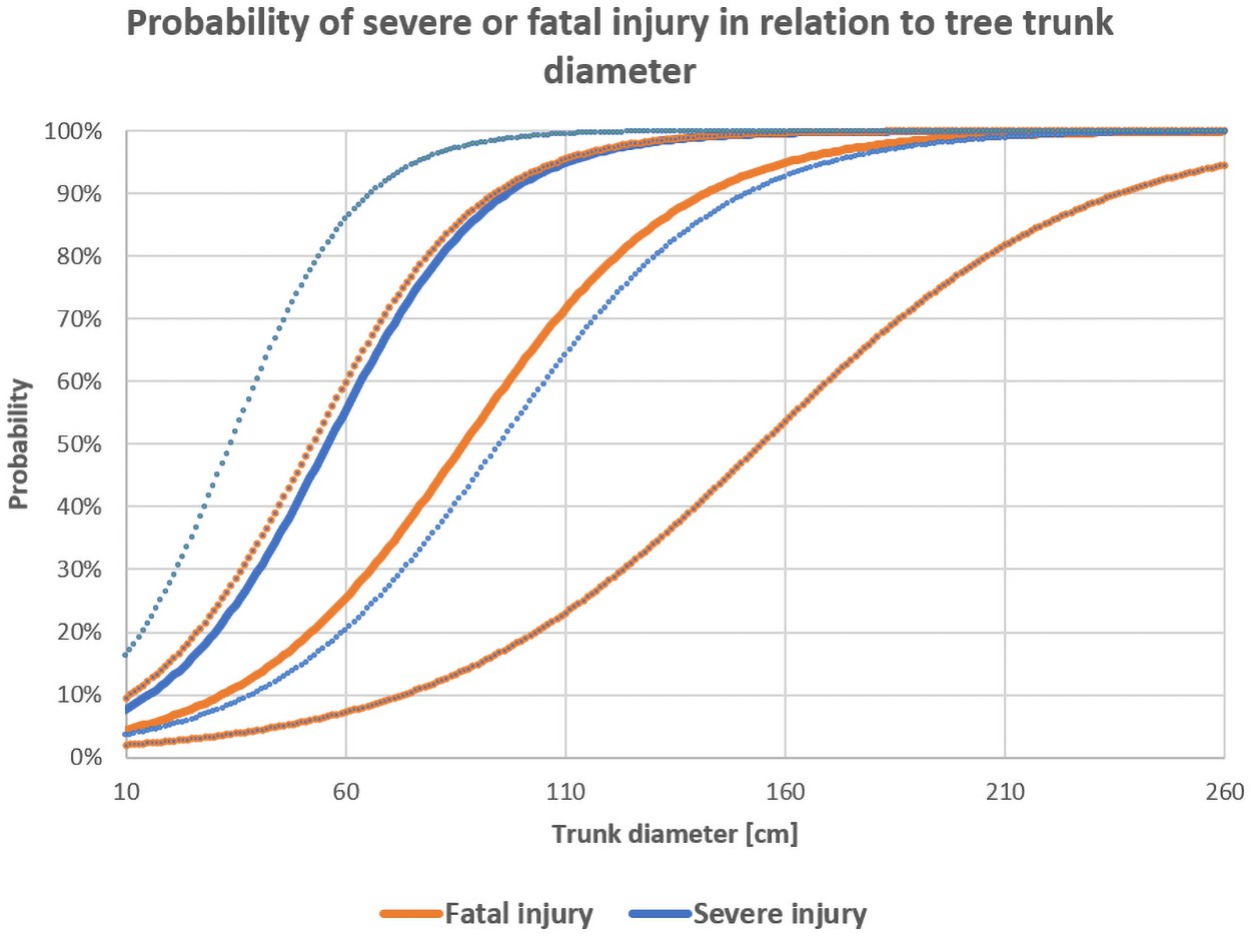


**Fig 2:**
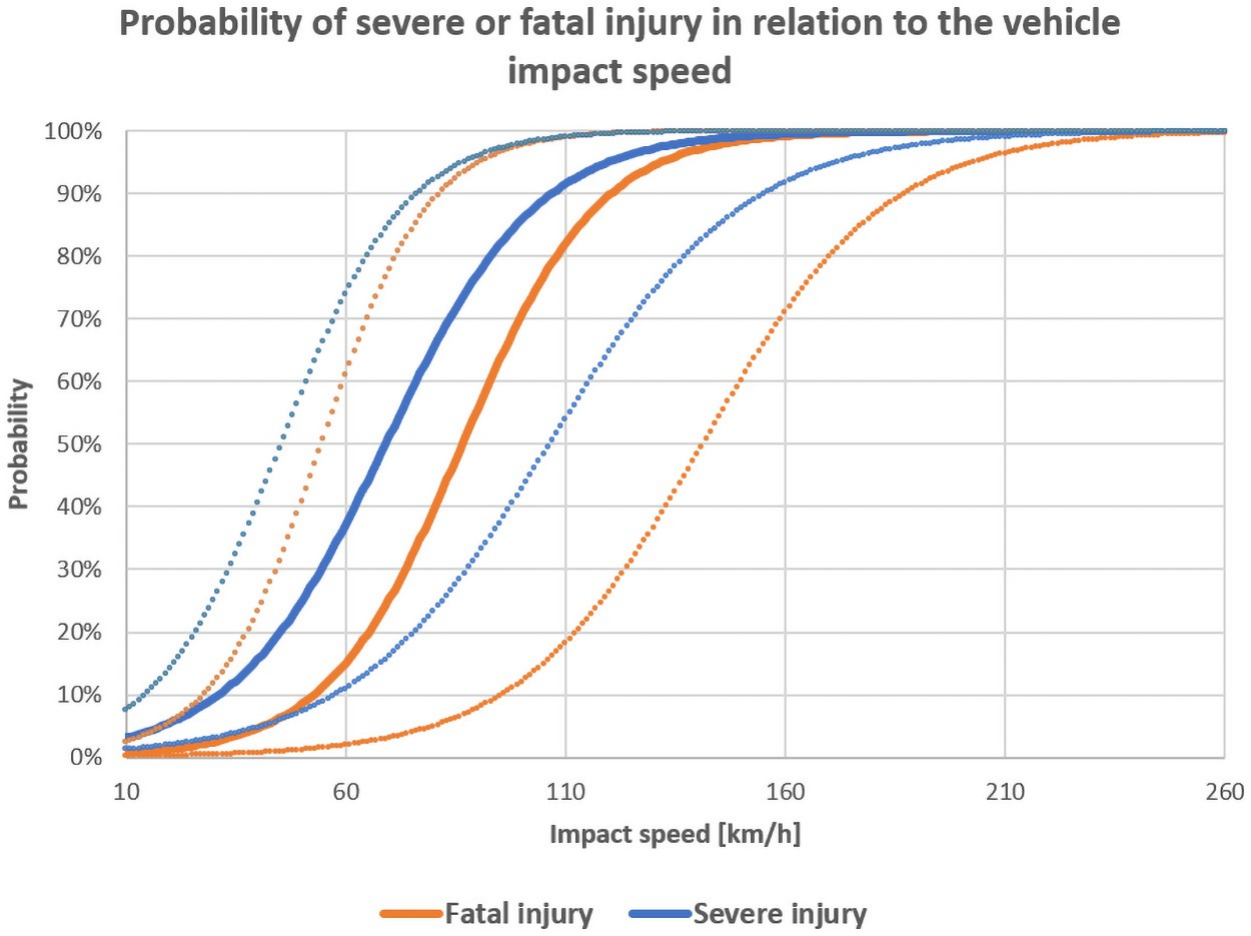


For the analysis of the risk of fatal or severe (fatal or serious) injury, the logistic regression analysis as a function of the impact speed or trunk diameter P(v), P(s) respectively was used. Using this analysis allows to predict dependency of the variable value between 2 possibilities (fatal/non-fatal, severe/non-severe) on selected continuous variable (impact speed, trunk diameter). The plot of the probability curve also contained confidence interval where the resulted curve lie with 95% probability. The probability equation:

$$P\left(v\right)=\frac{1}{1+e^{-\left(a+bv\right)}}$$ (7)

where *v* is the selected variable (impact speed, trunk diameter); *a*, *b* are parameters of the maximum likelihood. Parameter *a* determines the off-set of the logistic curve along the x-axis, parameter *b* determines the slope of the curve around the [-a/b; 1/2] [39, 44]. The resulting probability curves are illustrated in Figs 1 and 2.

The resulting functions of the impact speed are as follows:

$$P\left(v\_fatal\right)=\frac{1}{1+e^{-\left(-5,628+0,065x\right)}}$$ (8)

$$P\left(v\_severe\right)=\frac{1}{1+e^{-\left(-4,005+0,058x\right)}}$$ (9)

The resulting function of the trunk diameter is

$$P\left(s\_fatal\right)=\frac{1}{1+e^{-\left(-3,475+0,04x\right)}}$$ (10)

$$P\left(s\_severe\right)=\frac{1}{1+e^{-\left(-3,022+0,054x\right)}}$$ (11)

### Generalized linear mixed model

Due to correlation between explanatory variables we also considered a generalized linear mixed model. The outcome y is similarly categorical variable injury severity, grouping variable is every crash. For every crash participant were supposed 21 fixed effects plus a fixed intercept and random intercept.

We allow the intercept to vary randomly by each crash participant. Table 6 illustrates model results and individual model effects. The values of F reach significance with a p-value < 0.05, so there is a statistically significant difference between the means of the different levels of the severity injury variable. The resulting model contains significant variables: impact speed, trunk diameter, day period (estimated coefficients can be found in Table 7). Although the vehicle age is not a statistically significant variable in GLMM, it was kept in the following analysis for the purposes of the comparison with the previous model.

**Table 6:** ANOVA table for the overall model and the individual model effects.

	F statistic	Sig.
Corrected Model	2.352	.023
Impact speed	5.119	.008
Trunk diameter	4.114	.019
Day period	2.905	.050
Vehicle age	1.784	.173

**Table 7:** Parameter estimates.

Parameter		Coefficient	Std. Error	Sig	Exp(Coefficient)	95% confidence interval	
						Lower bound	Upper bound
Serious injury	Intercept	-7.542	2.186	.001	0.001	0.000	0.041
	Impact speed	.046	.023	.048	1.047	1.000	1.096
	Trunk diameter	.059	.029	.048	1.060	1.001	1.124
	day period = day	-1.435	.941	.166	.269	.042	1.742
	day period = night	0^b^	.	.	.	.	.
	Vehicle age	0.148	.085	.086	1.159	.979	1.372
Fatal injury	Intercept	-10.388	2.694	.000	0.000	0.000	0.006
	Impact speed	.083	.027	.003	1.087	1.029	1.147
	Trunk diameter	.088	.031	.005	1.092	1.027	1.160
	day period = day	-2.616	1.130	.023	.073	.008	.688
	day period = night	0^b^	.	.	.	.	.
	Vehicle age	.119	.090	.190	1.127	0.942	1.348

^a^. The reference category is: slight injury.

^b^. This parameter is set to zero—is used as reference category for comparison.

The obtained results show the values, significance tests, and confidence intervals for the individual model coefficients. GLMM estimates gave very similar estimates as multinomial logistic regression for all the parameters used in the analysis. Therefore, adding random effects for these parameters will not influence the model outcome. Neverthless Generalized Linear Mixed Models provide a good alternative for Generalized Linear Models.

## Discussion

As Van Treese II et al. [25] literature review showed, not many studies comprehensively analyzed a large number of factors associated with an increased risk of serious injury. Most of published studies used only selected factors. In addition, most of previous vehicle-tree collision analyses relied on Police data. Official police statistics allows to analyze various factors influencing the crash occurrence and injury severity. Official statistics could not contain all the data about the course of the crash, in most of the cases do not include vehicle driving speed or impact speed. As a substitution for the impact speed for the purpose of the analysis of factors influencing collision fatality, the information about speeding (exceeding the maximum speed allowance) has been used. E.g. in Liu and Subramanian [26] study as vehicle speeding status was considered crashes in which some of drivers involved in crash is charged with speeding-related offense or police officer indicate that racing, driving too fast or exceeding posted speed limit was factor related to the crash. The analysis without speed determination has been however very limited.

Most of the studies have been focused only on the modelling of relationship between the vehicle speed and injury severity or to the analysis frequency or type of crashes with fixed obstacles in relation to the vehicles driving speed. As described by Hu and Cicchino [45], lowering speed limits is a strategy that has been used to manage speeds in Canada, Europe and Australia. Mostly speed limit in the city area were reduced. Research in these countries has found reductions in speeds and crashes [45–47].

Some of the studies already analyzed the influence of selected fixed obstacle characteristic of road infrastructure on the injury severity. Zeigler [21] found that fatal crashes on rural roads were usually associated with larger trees (median 51 cm). Although the Zeigler study is older, in this paper, the tree trunk diameter value associated with fatal injuries is quite similar. However, the vehicle impact speed or driving speed was not analyzed in Zeigler study, because the used data did not allow speed determination.

There have been some limitations of the study because of the dataset characteristics. For the purpose of this study, the in-depth crash data was used. One of the limitation of this study could be the number of single vehicle—tree crashes used for modelling. In-depth crash studies are (in contrast to the more extensive national accident statistics) focused on detailed analysis of each investigated traffic crash. In-depth data provide a comprehensive view to the failure on the system infrastructure—human—vehicle related to crash and could serve to identify all the factors leading to crash occurrence or affect its consequences. In-depth database includes data related to infrastructure, vehicle, human failure and crash participants characteristics and their medical condition and also crash reconstruction data included impact speed. Approximately 3,000 of specific information are collected on each crash investigated within CzIDAS. Police reports are focused to the culprint assessment and mostly do not contain data about all factors related to the particular crash occurrence. CzIDAS is also not focused on a specific type of accidents, in contrast to some in-depth studies in which only selected types of accidents were investigated—e.g. the In-Depth Investigation of Motorcycle Accidents (MAIDS) project.

For the purpose of the analysis, only completely analyzed crashes (with crash reconstruction in simulation software) have been included to the study. For the purpose of this study, impact speed determined through Virtual Crash reconstruction in the technically acceptable range was used. The exact value of impact speed cannot be obtained, which may be the main limitation of this study. Currently Czech legislation system does not allow to use data from Event Data Recorders. Reconstruction is carried out on the basis of all the data obtained about the traffic crash—in particular the testimonials of the crash participants, vehicle damage, the plan of the crash site (including skid marks, brake marks, etc) and the polygon from the geodetic total station. Mutual collision of vehicles and their subsequent movement after collision into final positions after crash is solved by forward kinetic calculation.

## Conclusions

Every seventh dead in vehicle crashes in the Czech Republic is a consequence of vehicle-tree collision. The solution of this situation is not necessarily the tree removal. In some cases, it is possible to consider the installation of protective elements—e.g. guard rails.

Obtained results can help to efficiently direct countermeasures not only on the road infrastructure—e.g. speed reduction in selected locations with specified tree character. However, the emphasis should be also focused on driver behaviour. Renski et al. [48] pointed out that the presence of roadside trees could be based on the analysis an important consideration in the decision about speed limits. According to results of psychological studies (e.g. [49–51]), speed choice is associated with personality characteristics and risk-taking tendency. Speed is the factor, which influence the injury severity the most.

The tree trunk diameter is not a factor which could be influenced by the vehicle driver. However, the analysis carried out may help to focus the countermeasures on the transport infrastructure—to protect these trees with larger diameter with crash barriers, etc. Both significant factors—vehicle age and trunk diameter have an important role in terms of deformation energy. With regards to vehicle safety, it is necessary to maintain vehicle occupant survival space while maximizing absorption of the vehicle’s kinetic energy in the event of its collision. Older vehicles may experience gradual degradation of materials, whether corrosion of individual bodywork components. This results in a reduction in passive safety, a greater extent of vehicle damage, and in particular crew space, etc. For newer passenger cars, a lower proportion of serious crew injuries can be seen compared to older vehicles. The increase of the vehicle age about 10 years increased the probability of severe injury 4 times. The vehicle speed and vehicle age are the main factors which could be influenced by driver himself. With respect to the average age of vehicle fleet in the Czech Republic it would be also appropriate to emphasize the renewal of the vehicle fleet and more importantly to the technical condition of the vehicles. The results can also serve as an argument for adjusting national subsidy programs.
